# Converting Multi-Shell and Diffusion Spectrum Imaging to High Angular Resolution Diffusion Imaging

**DOI:** 10.3389/fnins.2016.00418

**Published:** 2016-09-14

**Authors:** Fang-Cheng Yeh, Timothy D. Verstynen

**Affiliations:** ^1^Department of Neurological Surgery, University of PittsburghPittsburgh, PA, USA; ^2^Department of Psychology and Center for the Neural Basis of Computation, Carnegie Mellon UniversityPittsburgh, PA, USA

**Keywords:** HARDI, multi-shell, diffusion spectrum imaging, connectome, connectomics, graph theoretical analysis, network measures

## Abstract

Multi-shell and diffusion spectrum imaging (DSI) are becoming increasingly popular methods of acquiring diffusion MRI data in a research context. However, single-shell acquisitions, such as diffusion tensor imaging (DTI) and high angular resolution diffusion imaging (HARDI), still remain the most common acquisition schemes in practice. Here we tested whether multi-shell and DSI data have conversion flexibility to be interpolated into corresponding HARDI data. We acquired multi-shell and DSI data on both a phantom and *in vivo* human tissue and converted them to HARDI. The correlation and difference between their diffusion signals, anisotropy values, diffusivity measurements, fiber orientations, connectivity matrices, and network measures were examined. Our analysis result showed that the diffusion signals, anisotropy, diffusivity, and connectivity matrix of the HARDI converted from multi-shell and DSI were highly correlated with those of the HARDI acquired on the MR scanner, with correlation coefficients around 0.8~0.9. The average angular error between converted and original HARDI was 20.7° at voxels with signal-to-noise ratios greater than 5. The network topology measures had less than 2% difference, whereas the average nodal measures had a percentage difference around 4~7%. In general, multi-shell and DSI acquisitions can be converted to their corresponding single-shell HARDI with high fidelity. This supports multi-shell and DSI acquisitions over HARDI acquisition as the scheme of choice for diffusion acquisitions.

## Introduction

Diffusion MRI offers a non-invasive way to map the structural connectivity of the human brain (Behrens et al., 2003, 2007; Wedeen et al., 2012), and several diffusion sampling schemes have been used to acquire the diffusion MRI data. The single-shell scheme, such as diffusion tensor imaging (DTI) (Basser et al., 1994; Basser and Pierpaoli, 1996) and high angular resolution diffusion imaging (HARDI) (Tuch et al., 2002; Tuch, 2004) remain the most popular diffusion MRI acquisition approaches, whereas multi-shell (Sotiropoulos et al., 2013) and diffusion spectrum imaging (DSI) (Wedeen et al., 2005) acquisition methods are becoming increasingly popular. For example, the Human Connectome Project (HCP) is acquiring diffusion MRI data using multi-shell (WU-Minn consortium; Sotiropoulos et al., 2013) and DSI schemes (USC-MGH consortium; Fan et al., 2016). Here we tested whether multi-shell and DSI data have conversion flexibility to be interpolated into corresponding single-shell HARDI data. Conversion flexibility, where data acquired from one scheme can be interpolated into another, would increase the analytical tractability of diffusion MRI data and facilitate comparing and aggregating results across studies with different acquisition approaches.

To explore this possibility, we used generalized q-sampling reconstruction (Yeh et al., 2010) to interpolate multi-shell and DSI data into their corresponding HARDI representation. These converted data sets are then compared with the original HARDI data acquired using a single-shell scheme. This comparison was conducted in both a single phantom study and several *in vivo* human studies. In the phantom study, HARDI, multi-shell, and DSI data were acquired. The multi-shell and DSI data were converted to a corresponding HARDI data set (hereafter referred to as the “converted HARDI” data set). A correlation analysis was conducted between the converted HARDI and the HARDI acquired from the MR scanner (termed “original HARDI” hereafter) to examine whether the converted HARDI can predict the original HARDI. In our *in vivo* study, we examined the correlation between their diffusion signals, anisotropy values, and diffusivity measurements. In addition, we further applied constrained spherical deconvolution (CSD; Tournier et al., 2007) to the converted and original HARDI and examined whether the angular error between the converted HARDI and the original HARDI. We also conducted tractography to generate connectivity matrices and determined their similarity using a correlation analysis. The network measures (Bullmore and Sporns, 2009) were also calculated using graph theoretical analysis to examine their difference.

## Materials and methods

### Signal interpolation

**Figure 1:**
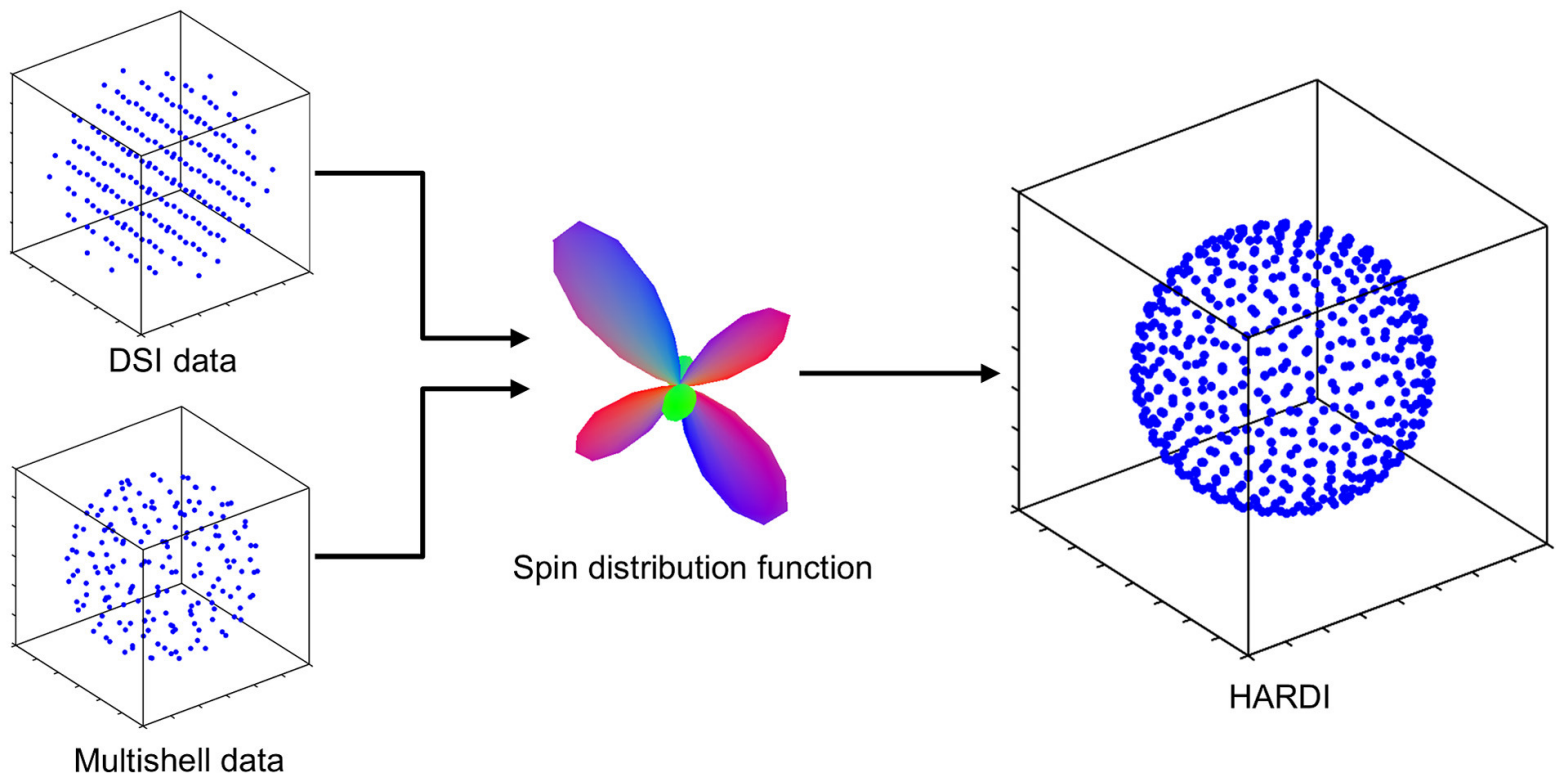
**The scheme conversion method uses the spin distribution function (SDF) to convert multi-shell or DSI data to their corresponding HARDI representation**. This is made possible by the linear relationship between the diffusion signals and the SDF provided by the generalized q-sampling reconstruction approach.

We interpolated DSI and multi-shell data into their corresponding HARDI using the generalized q-sampling method (Figure 1). Generalized q-sampling reconstruction provides a linear relation between diffusion MR signals and the spin distribution function (SDF; Yeh et al., 2010). This linear relation enables a direct conversion between SDFs and diffusion signals acquired from single-shell (HARDI), multi-shell, and grid (DSI) schemes. SDF measures the density of diffusing water at different orientation and is thus a measurement of spin density. It is thus different from the diffusion orientation distribution function (dODF), which is normalized as a probability density function and “unit-free.” It is also different from fiber orientation distribution function (fODF) calculated from spherical deconvolution, which represents the volume fraction of the fiber distribution and is a fractional measurement.

Studies have shown that the SDFs from different schemes present a consistent pattern (Yeh et al., 2010, 2011; Yeh and Tseng, 2013), and thus we can make use of the SDF to convert diffusion signals from one sampling scheme to another. DSI or multi-shell data can be converted to a common SDF and the linear relation between SDF and the HARDI signals will allow for estimating the corresponding HARDI representation by solving the inverse problem using constraint optimization.

To illustrate this idea, we start with the generalized q-sampling reconstruction that is based on the linear relation between the diffusion MRI signals and the spin distribution function (SDF).

(1) $$\begin{array}{l} \Psi =\text{A}\cdot \text{w} \end{array}$$

where Ψ is a diffusion ODF vector, and w is a vector of diffusion MRI signals, and its *i*-th dimension represents the diffusion signal acquired by a *b*-value of *b*_*i*_ and diffusion gradient direction (b-vector) of ${\hat{b}}_{i}$. **A** is a matrix, and its element, A_*i, j*_, at row *i* and column *j* is defined as follows:

(2) $$\begin{array}{l} {\text{A}}_{i,j}=\text{sinc}\left(\sigma \sqrt{6D\cdot |{\text{b}}_{i}|}\cdot \langle \frac{{\text{b}}_{i}}{\left|{\text{b}}_{i}\right|},{\hat{\text{u}}}_{j}\rangle \right) \end{array}$$

where σ is a length ratio that controls the detection radius of the diffusion. *D* is the diffusion coefficient of free water diffusion and û_*j*_ is a unit vector representing the *j*-th direction of the diffusion ODF. Using Equation (1), we can convert the DSI or multi-shell signals, **w**, to their corresponding HARDI representation, **w**_*h*_, by equalizing their SDFs.

(3) $$\begin{array}{l} {\text{A}}_{h}\cdot {\text{w}}_{h}=\text{A}\cdot \text{w}\;s.t.\;{\text{w}}_{h}\geq 0 \end{array}$$

where A_*h*_ is a matrix defined by an HARDI b-table, and **w**_*h*_ is the corresponding HARDI representation to estimate. Equation (3) formulates the conversion of the MRI signals as an inverse problem, and we can construct an over-determined equation (more equations than unknowns) by assigning more sampling directions in SDF than in HARDI. Equation (3) can be solved by using the Tikhonov regularization.

(4) $$\begin{array}{l} {\text{w}}_{h}\approx {\left({\text{A}}_{h}^{T}{\text{A}}_{h}+\lambda \text{I}\right)}^{-1}{\text{A}}_{h}^{T}\text{A}\cdot \text{w} \end{array}$$

where λ is a regularization parameter and we chose the smallest possible value that resulted in more than 99% of the estimated HARDI representation being positive within the brain mask. This loosens the constraint for Equation (3) and provides a quick estimation for the constraint optimization. The conversion routine was implemented in DSI Studio (http://dsi-studio.labsolver.org), a publicly available and open source tool. The source code for scheme conversion can be found at https://github.com/frankyeh/DSI-Studio (search for “SchemeConverter”).

### Correction for gradient nonlinearity

The nonlinearity of diffusion gradients can induce a prominent image distortion that alters the effective *b*-values (Bammer et al., 2003; Sotiropoulos et al., 2013). This can be corrected using the nonlinear terms of the magnetic field obtained from gradient coils (Jovicich et al., 2006). The multi-shell data set from the WU-Minn consortium includes a 3-by-3 gradient deviation matrix **G** for each voxel to estimate the effective gradient direction and strength. This matrix can be directly embedded in the element of matrix A to account for the effect of gradient nonlinearity:

(5) $$\begin{array}{l} {\text{A}}_{i,j}=\text{sinc}\left(\sigma \sqrt{6D\cdot |{\text{Gb}}_{i}|}\cdot \langle \frac{{\text{Gb}}_{i}}{\left|{\text{Gb}}_{i}\right|},{\hat{\text{u}}}_{j}\rangle \right) \end{array}$$

Equation (5) can replace Equation (2), and the converted HARDI representation already considers the gradient nonlinearity.

### Diffusion phantom

**Figure 2:**
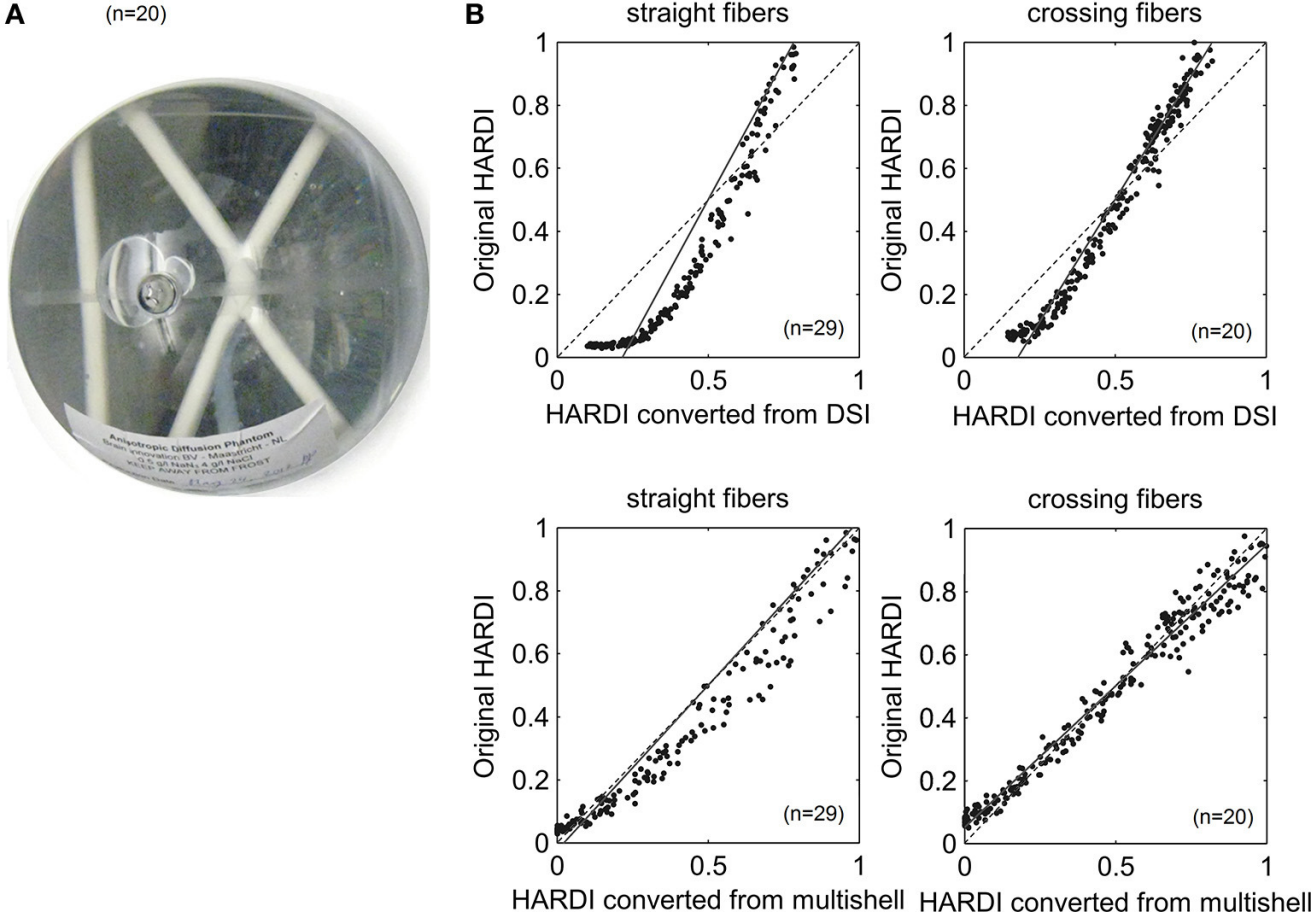
**(A)** The layout of the anisotropic diffusion phantom consists of one straight fiber bundle and a pair of crossing bundles. **(B)** Scatter plots showing a signal correlation between the converted HARDI and the original HARDI in our phantom study. The dotted lines indicate identity. The converted HARDI was calculated from the multi-shell or DSI data, whereas the original HARDI was acquired from the MR scanner. All comparisons had a high correlation coefficient (*r* > 0.9), suggesting that the converted HARDI data strongly predict the original HARDI images.

An anisotropic diffusion phantom (Brain Innovation, Maastricht, Netherlands; Pullens et al., 2010) was scanned using a 3T Siemens Verio scanner (Siemens, Erlangen, Germany) in the Scientific Imaging and Brain Research Center at Carnegie Mellon University. The maximum gradient strength was 45 mT/m. The phantom consists of one straight fiber bundle and a pair of crossing fiber bundles, as shown in Figure 2A. Each bundle contains 10,000 polyester yarns (KUAG Diolen, 22 dtex 18) and each yarn is made up of 18 fibers. The production process and phantom property are detailed in the corresponding reference above. The acquisition parameters for HARDI, multi-shell, and DSI schemes are listed in Table 1. The images were acquired using the same spatial parameters: the field of view was 288 × 288 mm, the matrix size was 96 × 96, the slice thickness was 3.0 mm (no gap), resulting in a voxel size of 3.0 × 3.0 × 3.0 mm. The multi-shell and DSI data were converted to 256-direction HARDI using a regularization parameter of 0.05. Since the actual values of the diffusion signals can be scaled due to scanner settings, to facilitate comparison, both converted and original HARDI were multiplied by a scaling constant to equalize the mean of the overall signals. To exclude background noise, we selected two regions of interest (ROIs), one placed on the straight fibers (29 voxels) and another placed at the center of the crossing fibers (20 voxels). The diffusion signals at these two ROIs were averaged to increase the signal to noise ratio. Each diffusion weighted image had one average signal for the straight fibers, and one average signal for the crossing fibers. A total of 256 average values (one average value from each diffusion-weighted image) were calculated for original HARDI. The same processing procedures were applied to the HARDI converted from DSI and multi-shell data. A correlation analysis between the converted and original HARDI was conducted to examine whether the converted HARDI can predict original HARDI.

**Table 1:** **Summary of diffusion scans in phantom and ***in vivo*** study**.

**Scheme**	***b*-value (s/mm^2^)**	**Number of diffusion gradients**	***TR* (ms)**	***TE* (ms)**
**PHANTOM STUDY**				
DSI	400~4000	257	1800	135
Multi-shell	1500/3000	30/64	2100/2500	135/143
HARDI	3000	256	2500	143
***IN VIVO*** **STUDY**				
DSI	400~4000	202	7200	144
Multi-shell	1500/3000	30/64	5500/6300	101/121
HARDI	4000	252	7200	133

### *In vivo* experiment

We used publicly available data from Advanced Biomedical MRI Lab at National Taiwan University Hospital (http://dsi-studio.labsolver.org/download-images). The data include HARDI, multi-shell, and DSI data acquired on a 25-year-old male subject using a 3T MRI system (Tim Trio; Siemens, Erlangen, Germany). The maximum gradient strength was 40 mT/m. A 12-channel coil and a single-shot twice-refocused echo planar imaging (EPI) diffusion pulse sequence was used to acquire HARDI, multi-shell, and DSI data on the same subject, as summarized in Table 1. The HARDI, multi-shell, and DSI data were acquired using the same spatial parameters: the field of view was 240 × 240 mm, the matrix size was 96 × 96, the slice thickness was 2.5 mm (no gap), and the number of the slices was 40 to cover the cerebral cortex, resulting in a voxel size of 2.5 × 2.5 × 2.5 mm. The multi-shell and DSI data were converted to 252-direction HARDI using a regularization parameter of 0.05. Both converted and original HARDI were scaled by a constant to equalize the mean of the overall signals. The JHU white-matter tractography atlas (Laboratory of Brain Anatomical MRI, Johns Hopkins University) was used to segment the corpus callosum (1383 voxels), cerebral peduncle (229 voxels), coronal radiata (1385 voxels), internal capsule (677 voxels), and cingulum pathways (286 voxels). Unlike what was done in the phantom study, we did not average the signals of each region because the containing fibers may not have the same orientation. A correlation analysis between converted and original HARDI signals was conducted to examine whether the converted HARDI representation can predict those of the original HARDI. Furthermore, we calculated generalized fractional anisotropy (GFA), fractional anisotropy (FA), and mean diffusivity (MD) from the converted and original HARDI. The GFA was calculated from q-ball imaging (Tuch, 2004), whereas FA was estimated using DTI, and MD was calculated by averaging the axial and radial diffusivities calculated from the tensor model. The correlation of the indices between converted and original HARDI was analyzed at voxels with a different signal-to-noise ratio (SNR). The SNR was estimated using the original HARDI data.

We also calculated the fODF from the converted HARDI and examined whether they presented the same pattern as those from the original HARDI. The converted and original HARDI were reconstructed using constrained spherical deconvolution (MRtrix, www.nitrc.org/projects/mrtrix) to obtain fODFs. As suggested in the MRtrix's user document, the response function was estimated using voxels with FA value greater than 0.7 and a maximum harmonic order of 8 were used. The calculated fODFs were used to resolve fiber orientations, and the angular error between converted HARDI and the original HARDI was calculated to examine the consistency of fODFs across schemes.

### Graph theoretical analysis

A deterministic fiber tracking algorithm (Yeh et al., 2013) was applied to obtain a total of 5000 tracks using an anisotropy threshold that covered the white matter region. The angular threshold was 60°. The connectivity matrices were calculated using the Automated Anatomical Labeling (AAL) atlas (Tzourio-Mazoyer et al., 2002). A total of 116 regions were nonlinearly transformed to the subject space in DSI Studio. The fiber count was used as the matrix entry. A binary form of the connectivity matrices was obtained by a threshold of 0.1% of the maximum matrix value. Network measures, such as characteristic path length, global efficiency, clustering coefficient, and betweenness centrality, were calculated using graph theoretical analysis (Bullmore and Sporns, 2009). The differences of the measurements were computed between the converted HARDI and the original HARDI to quantify the discrepancy.

### Human connectome project data

A multi-shell data set was selected from the WU-Minn consortium (subject# 113619) and a DSI data set was from USC-MGH consortium. The WU-Minn multi-shell data were acquired in a Siemens 3T Skyra scanner using a 2D spin-echo single-shot multiband EPI sequence with a multi-band factor of 3 and monopolar gradient pulse (Sotiropoulos et al., 2013). The spatial resolution is 1.25 mm isotropic. *TR* = 5500 ms, *TE* = 89 ms. The *b*-values were 1000, 2000, and 3000 s/mm^2^. The total number of diffusion sampling directions was 270. The total scanning time was approximately 55 min. The multi-shell data were converted to HARDI using a regularization parameter of 0.05 and a *b*-value of 4000 s/mm^2^. The diffusion gradient nonlinearity was corrected using Equation (5). The converted HARDI was analyzed using CSD implemented in MRtrix (http://www.nitrc.org/projects/mrtrix/). To examine the angular resolution of our converted HARDI, we compared the CSD results with the ball-and-sticks model (Behrens et al., 2003), the recommended analysis method for the WU-Minn HCP data (Sotiropoulos et al., 2013). The angular deviation between fiber orientations was calculated to examine whether their orientations are substantially close to each other. The FSL's *bedpostx* program was used to resolve a maximum of 3 fibers per voxel. All default parameters were used. The resolved fiber orientations were compared with those resolved by CSD applied to the converted HARDI.

The USC-MGH DSI data were acquired in a Siemens 3T Connectome scanner equipped with a 300 mT/m gradient system (McNab et al., 2013). A 2D spin-echo EPI sequence was used to acquire diffusion images. The spatial resolution is 1.5 mm isotropic. *TR* = 4200 ms, *TE* = 53 ms. The maximum *b*-value was 15,000 s/mm^2^, and the total number of diffusion sampling directions was 515. The DSI data were converted to HARDI using a regularization parameter of 0.05 and a *b*-value of 4000 s/mm^2^. The converted HARDI was analyzed using CSD implemented in MRtrix (http://www.nitrc.org/projects/mrtrix/). The CSD results were compared with DSI reconstruction.

## Results

### Diffusion signals

Figure 2B shows the scatter plots of the diffusion MRI signals from the converted HARDI and original HARDI data sets. The dotted lines indicate identity. The regression equation and correlation coefficient are listed in Table 2. The converted HARDI data are strongly correlated with the original acquired HARDI data in the phantom. The high correlation coefficient (>0.9) suggests that the converted HARDI is a good predictor of the original HARDI. It should be noted that the HARDI converted from the DSI data has a positive x-intercept. This can be due to differences in the *b*-value and the effective diffusion time.

**Table 2:** **Results of correlation analysis in the phantom and ***in vivo*** study**.

**Original data**	**Location**	**Regression line**	**Correlation coefficient**
**PHANTOM**			
DSI	Straight fibers	*y* = 1.7621 × −0.3810	0.9576
DSI	Crossing fibers	*y* = 1.5545 × −0.2772	0.9766
Multi-shell	Straight fibers	*y* = 1.0467 × −0.0234	0.9773
Multi-shell	Crossing fibers	*y* = 0.8970 × +0.0515	0.9866
***IN VIVO***			
DSI	Corpus callosum	*y* = 1.1045 × −0.0435	0.8251
DSI	Cerebral peduncle	*y* = 0.9628 × +0.0303	0.7912
DSI	Coronal radiata	*y* = 0.9595 × +0.0225	0.6777
DSI	Internal capsule	*y* = 0.9756 × +0.0258	0.7338
DSI	Cingulum	*y* = 0.9067 × +0.0099	0.6408
Multi-shell	Corpus callosum	*y* = 0.8335 × +0.0795	0.8476
Multi-shell	Cerebral peduncle	*y* = 0.7951 × +0.1128	0.7996
Multi-shell	Coronal radiata	*y* = 0.7812 × +0.1148	0.7207
Multi-shell	Internal capsule	*y* = 0.8073 × +0.1228	0.7617
Multi-shell	Cingulum	*y* = 0.7317 × +0.0925	0.6735

**Figure 3:**
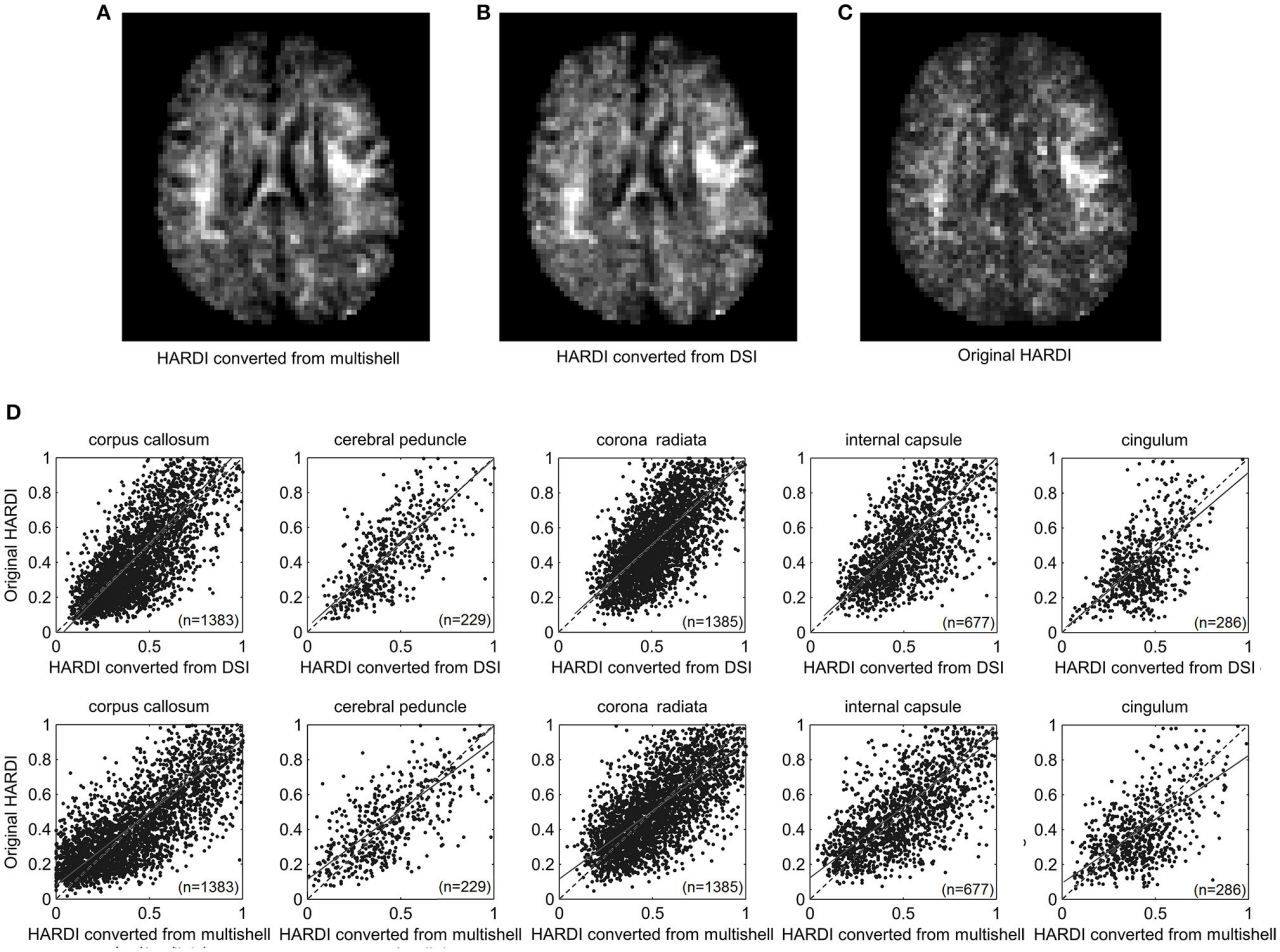
**The HARDI images converted from (A) a multi-shell scheme and (B) a DSI scheme compared with (C) the original HARDI images from the scanner**. The converted HARDI images show a signal pattern similar to the original HARDI acquired from the MRI scanner. **(D)** The scatter plots show the signal correlation between the converted HARDI and the original HARDI in our *in vivo* study. The converted HARDI data correlates well with the original HARDI images (*r* > 0.6).

Our *in vivo* experiment also showed a high correlation between the converted HARDI and the original HARDI (Figure 3). The dotted lines indicate identity. The HARDI images converted from multi-shell (Figure 3A) and DSI (Figure 3B) and the original HARDI image acquired from the MRI scanner (Figure 3C) share high similarity in the axial views of the centrum semiovale. The converted HARDI images (Figures 3A,B) show a signal intensity pattern consistent with the original HARDI image (Figure 3C). The hyperintensity regions are consistent in the converted and original HARDI. Figure 3D shows the scatter plots of the diffusion MRI signal from the HARDI converted from DSI and multi-shell data against those from the original HARDI at different brain regions. The regression equations and correlation coefficients are listed in Table 2. The correlation coefficients range from 0.67 to 0.84, suggesting a strong similarity in signal values regardless of the brain regions. The HARDI converted from DSI shows regression equations with coefficients around 1.0 and intercepts around 0. However, the HARDI converted from multi-shell results in regression equations with coefficients around 0.8 and intercepts around 0.1. The bias can be attributed to the *b*-value difference between the multi-shell acquisition (*b* = 1500 and 3000 s/mm^2^) and the original HARDI (*b* = 4000 s/mm^2^). The multi-shell data were acquired by lower *b*-value and the signals were higher, resulting in biased coefficients.

### Anisotropy and diffusivity

**Figure 4:**
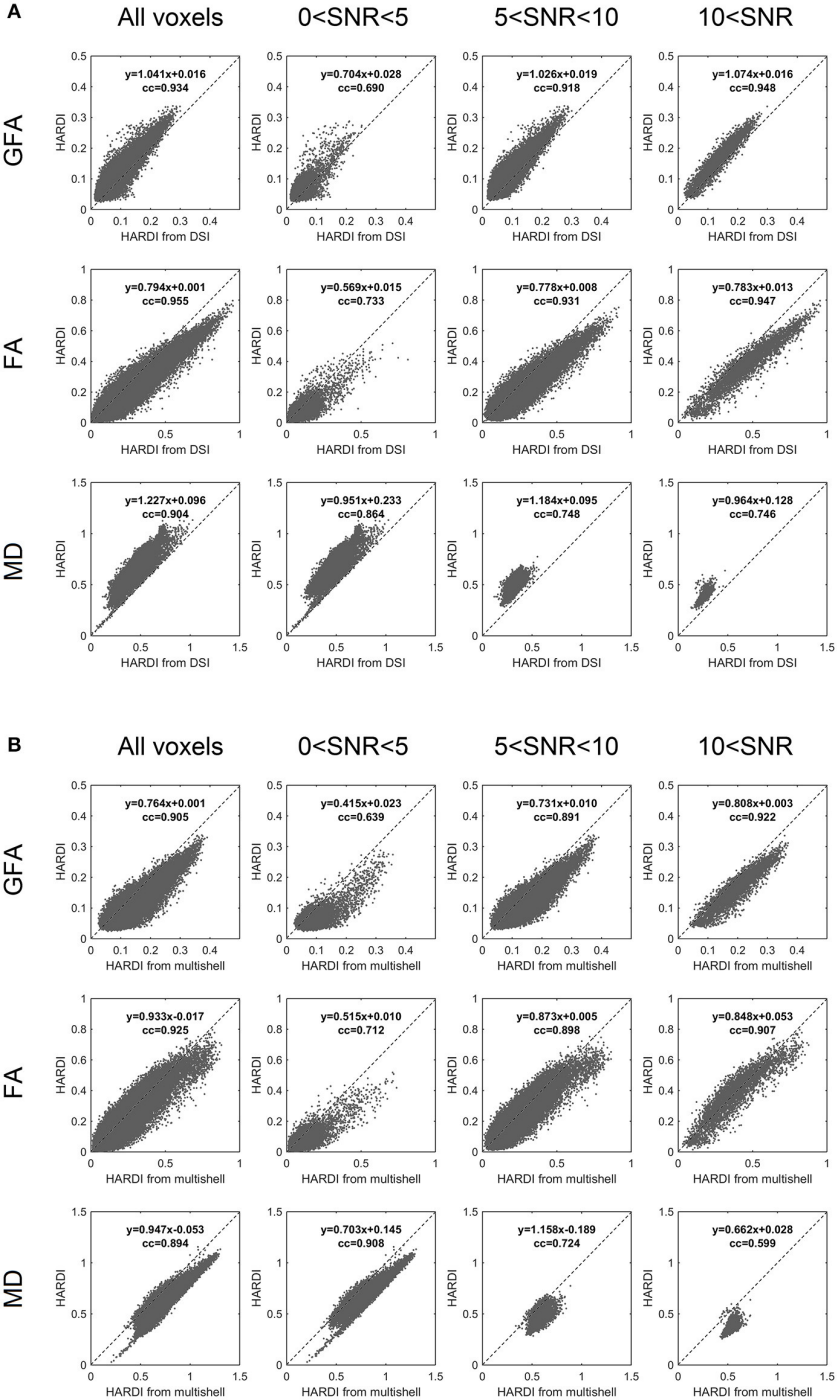
**GFA, FA, MD measurements between (A) HARDI converted from DSI and original HARDI and between (B) HARDI converted from multishell and original HARDI**. The dotted lines indicate identity. The dotted lines indicate identity. identity. For both DSI and multishell schemes, the converted HARDI shows high correlation coefficients of GFA, FA, and MD around 0.9 with those of the original HARDI. The low SNR voxels also present moderate to good correlation, suggesting that HARDI converted from DSI and multishell can provide highly predictive GFA, FA, and MD measurements.

The anisotropy and diffusivity measurements calculated from the converted HARDI strongly predict those of the original HARDI (Figure 4). The analysis was conducted across all voxels (within the brain mask) as well as voxels with different SNRs estimated from original HARDI so as to understand how the SNR affects the correlation. The results on all voxels show high correlation coefficients around 0.8 for all indices. At a higher SNR, the converted HARDI reliably predicts the anisotropy measurements. The correlation coefficient between MD at high SNR is lower because the MD is limited to a narrow dynamic range with no obvious linearity; however, the lower correlation coefficient value does not indicate poor predictivity. On the contrary, the scatter plots show how converted HARDI still reliably predicts the same MD value within the same dynamic range. It is noteworthy that the HARDI converted from DSI shows overall higher FA values than those of the original HARDI while the HARDI converted from multi-shell data shows a consistent higher GFA values. This bias is expected as studies have shown that the anisotropy and diffusivity measurements are *b*-value dependent (Hui et al., 2010; Papinutto et al., 2013).

### Fiber orientations

**Figure 5:**
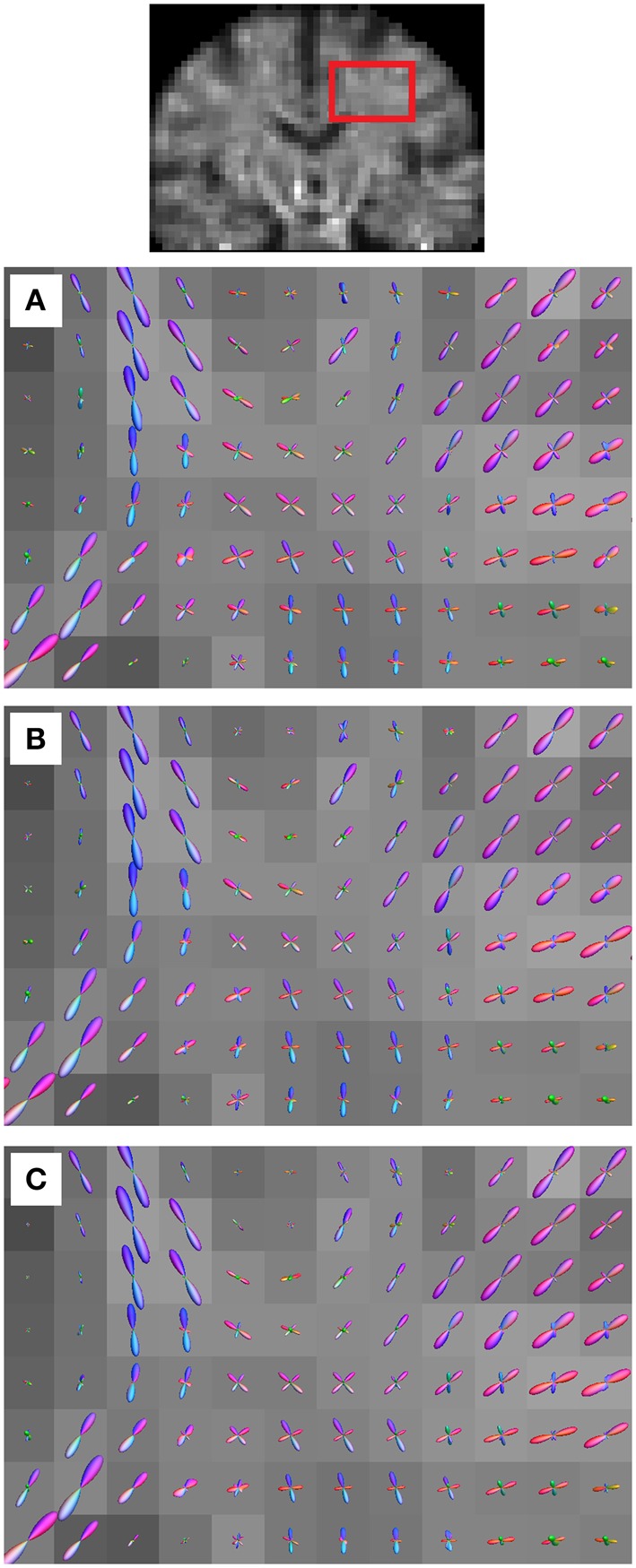
**The results of CSD applied to (A) the HARDI converted from the multi-shell data, (B) the HARDI converted from DSI data, and (C) the original HARDI data**. The fiber orientation distribution functions (fODFs) show similar patterns and comparable angular resolution.

We also found that the fODFs from the converted HARDI data are consistent with the fODFs from the original HARDI data. Figure 5 shows the results of CSD applied to the HARDI converted from the multi-shell scheme (Figure 5A), the HARDI converted from the DSI scheme (Figure 5B), and the original HARDI (Figure 5C). The fODFs are presented in a coronal view focusing on the same slice covering the central semiovale. The fODFs of the converted and original HARDI present very similar shapes in these voxels. The crossing fibers formed by corpus callosum (horizontal) and corticospinal tracts (vertical) can be resolved using converted HARDI though the converted HARDI was calculated using fewer diffusion sampling directions.

**Figure 6:**
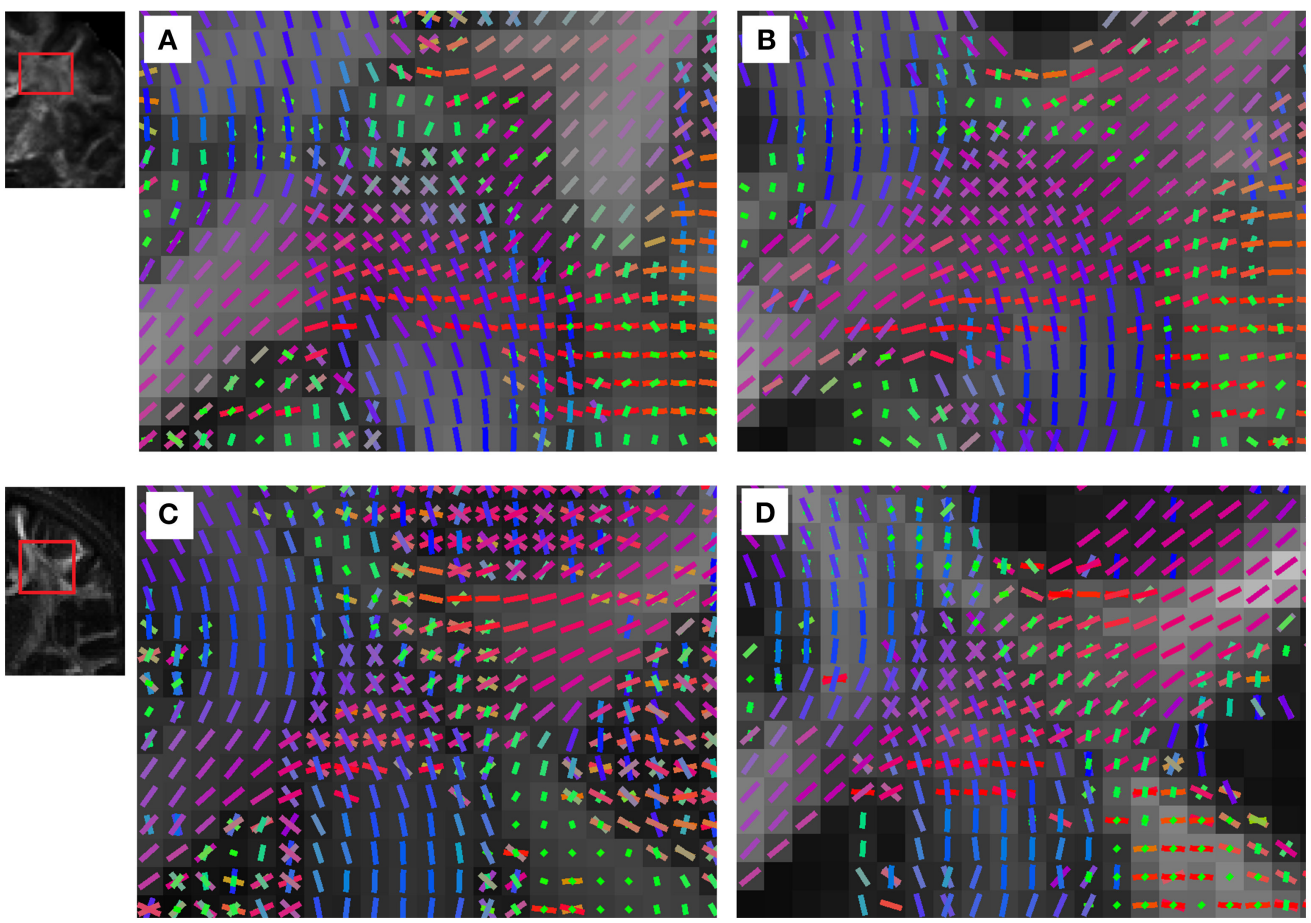
**The fiber orientations resolved by (A) CSD applied to the multi-shell data of the WU-Min consortium using our scheme conversion method, (B) ball-and-sticks model applied to the same WU-Min consortium data, (C) CSD applied to the DSI data of the USC-MGH consortium using our scheme conversion method, and (D) DSI reconstruction applied to the same USC-MGH consortium data**. The data converted from and multi-shell and DSI achieve a comparable angular resolution, and the crossing fibers can be readily resolved.

A more consistent pattern of results is seen in the multi-shell and DSI data from the two HCP consortiums. The converted HARDI were analyzed using CSD, and the resolved fiber orientations were compared with those from the ball-and-sticks model and DSI applied to the original data. The fiber orientations resolved by CSD applied to the HARDI converted from the WU-Minn consortium multi-shell data are shown in Figure 6A, whereas the fiber orientations resolved by ball-and-sticks model applied to the same data are shown in Figure 6B. The underlying maps are the maximum values of the fODFs and dODFs calculated from CSD and DSI, respectively. The figures are coronal views focused on the centrum semiovale, whereas the horizontally going corpus callosum intersects with the vertically going corticospinal tracks and the superior longitudinal fasciculus that passes in-plane. The CSD and ball-and-sticks models resolve a similar pattern of crossing fibers, suggesting that the HARDI converted from the multi-shell scheme can achieve a comparable angular resolution. Likewise, the fiber orientations resolved by CSD applied to the HARDI converted from the USC-MGH consortium DSI data are shown in Figure 6C, whereas the fiber orientations resolved by DSI reconstruction applied to the same data are shown in Figure 6D. The figures are also coronal views focused on the centrum semiovale. The CSD and DSI resolve a similar pattern of crossing fibers. The converted HARDI datasets displayed substantially more false positives fibers in the ventricles than the original multi-shell and DSI acquisitions. This may be due to a limitation of CSD reconstruction to handle the partial volume of free diffusion (Dell'Acqua et al., 2009).

**Figure 7:**
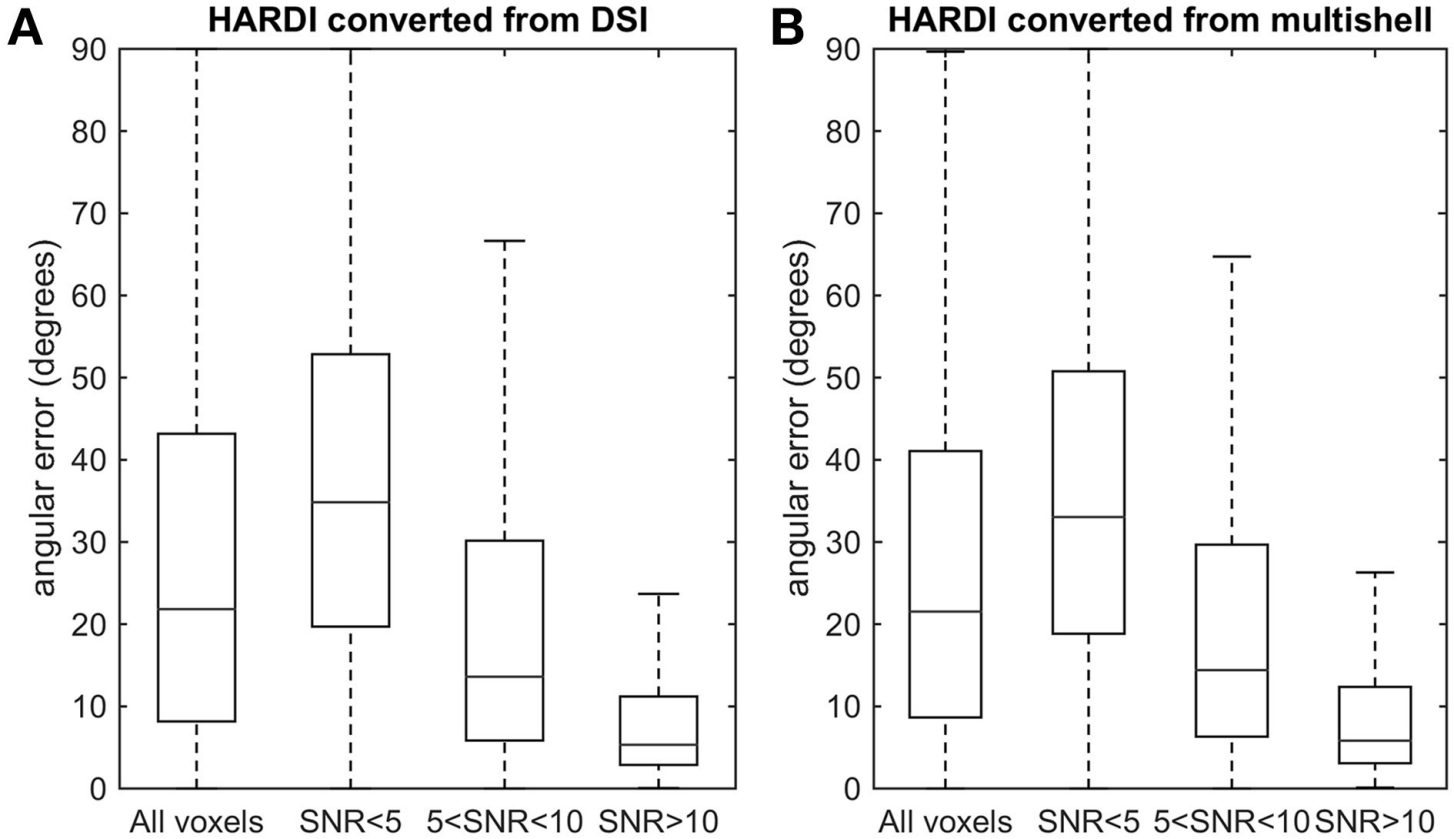
**Boxplots showing the angular error between (A) HARDI converted from DSI and the original HARDI and (B) HARDI converted from multishell and the original HARDI**. Overall, the median of the angular error is around 20°. At voxels with SNR greater than 5, the angular error is substantially lower than the limit an orientation distribution function, suggesting that the fiber orientations calculated from the converted HARDI are sufficiently close to those of the original HARDI.

To quantify the similarity between the fiber orientations, we calculated the angular error between the converted and the original HARDI (Figure 7). The analysis was conducted on the data from our *in vivo* experiment. The primary fiber orientations were determined from the peak orientation of the fODFs. The angular error (in degrees) was calculated for voxels with different SNR. In Figure 7A, the HARDI converted from DSI shows angular errors between 10° and 40° (the 1st and 3rd quartiles). For voxels with SNR greater than 5, the average angular error was 20.7°, which is smaller than angular resolution of the deconvolution methods (around 15°~30°) (Tournier et al., 2008; Yeh and Tseng, 2013). In Figure 7B, the HARDI converted from multi-shell data presents a similar pattern of results. The average angular error was also 20.7°. The low angular error suggests that the fODFs calculated from converted HARDI are consistent with the original HARDI and that the converted HARDI can achieve an angular resolution comparable to the original HARDI.

### Connectivity matrix and network measures

**Figure 8:**
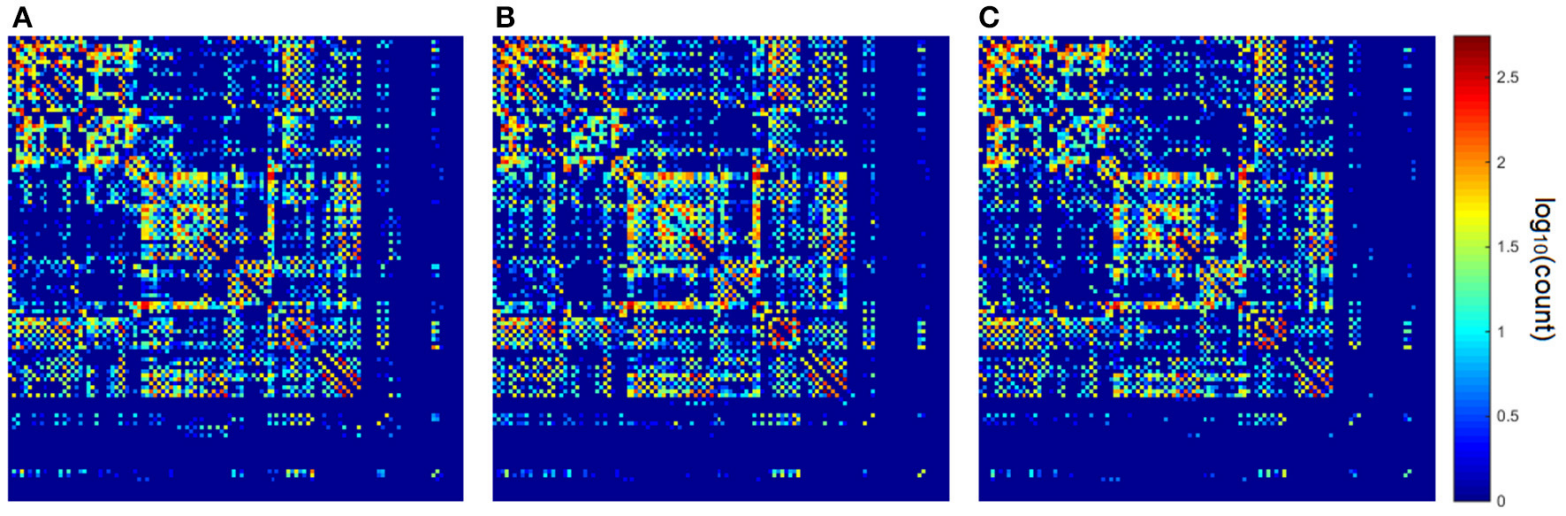
**The connectivity matrices calculated from (A) the HARDI converted from the multi-shell data, (B) the HARDI converted from DSI data, and (C) the original HARDI data**. Graph theoretical analysis shows that less than 2% difference in network topology and around 4~7% difference in nodal measures.

The similarity in voxel-wise fiber angle estimates suggests that these methods should produce highly similar tractography results. To determine this, we compared the whole-brain connectivity results to a set white matter areas using deterministic tractography (see Materials and Methods). Figure 8 shows the connectivity matrix calculated using the HARDI converted from the multi-shell scheme (Figure 8A), the HARDI converted from the DSI scheme (Figure 8B), and original HARDI (Figure 8C). We further conducted graph theoretical analysis to examine the consistency between the network topology. The major network measures calculated are listed in Table 3. The network topology measures such as characteristic path length and global efficiency show less than 2% difference, whereas the average nodal measures such as clustering coefficient and centrality show a difference around 4~7%. This result suggests the network topology remains high consistency across schemes, whereas the local nodal pattern such as clustering coefficient and betweenness centrality may differ more.

**Table 3:** **Network measures calculated from converted HARDI and original HARDI**.

	**HARDI from DSI**	**Change (%)**	**HARDI from multishell**	**Change (%)**	**Original HARDI**
Characteristic path length	1.572	−0.581	1.582	0.064	1.581
Global efficiency	0.719	1.175	0.712	0.238	0.711
Average cluster coefficient	0.607	6.909	0.589	3.860	0.567
Average betweenness centrality	49.397	4.677	50.293	6.576	47.190

## Discussion

There has been a long-standing debate about whether acquisition using multiple *b*-values is more efficient than single-shell acquisitions (Kuo et al., 2008; Tournier et al., 2011; Sotiropoulos et al., 2013; Daducci et al., 2014). Currently, single-shell acquisition schemes still remain the most popular diffusion MRI acquisition approach in clinical and research scanners (Tournier et al., 2011; Le Bihan and Johansen-Berg, 2012; Abhinav et al., 2014). While identifying the true optimal acquisition scheme is still under active investigation, it is crucial to investigate how diffusion characteristic and network measures changes due to different diffusion sampling schemes. Here we show that schemes with multiple *b*-values have conversion flexibility. The diffusion signals converted from DSI and multi-shell acquisition strongly predict those of the original HARDI in our phantom and *in vivo* experiment. The high predictive power was also observed for common diffusion indices, such as fractional anisotropy and diffusivity. The fODF, connectivity matrix, and network measures all show a highly similar profile between converted HARDI and the original HARDI. Taken together, our results show that multi-shell and DSI acquisitions can be converted to their corresponding single-shell HARDI and be used to supplement the collection of original HARDI data. Since it is more difficult to convert single-shell data into a multi-shell or DSI representation, the asymmetrical direction of the conversion suggests that acquiring diffusion MRI data with a single-shell scheme inherently limits analytical accessibility. Moreover, while multi-shell and DSI can distinguish partial volume of free water, the converted HARDI cannot effectively remove the effect (shown in Figure 6). This provides crucial evidence that, until a true optimal sampling scheme is identified, the diffusion community should adopt acquisition schemes with multiple *b*-values, such as multi-shell or DSI, over single-shell schemes such as DTI and HARDI.

While the current results show that the diffusion scheme can be converted from DSI and multi-shell to single-shell HARDI, there are some limitations to this approach that should be considered before applying it to existing data sets. As shown in our phantom and *in vivo* study, the diffusion images acquired from low *b*-value may have a bias if they are converted to a high *b*-value scheme. The bias can be observed in the anisotropy and diffusivity measurements, though the conversion still resulted in a reasonably high correlation with the original HARDI data set. This is due to the fact that low *b*-value acquisitions are more sensitive to nonrestricted diffusion, whereas high *b*-value acquisitions are more sensitive to restricted diffusion (Callaghan, 1991). Different *b*-values may also have different diffusion times and diffusion encoding durations. Unfortunately, information on the diffusion time and diffusion encoding duration was not supplied by the manufacturer or the spin-echo sequence used. Consequently, the diffusion indices derived from different *b*-values cannot be directly compared. In addition, the conversion is one-way, i.e., we cannot use the SDF to convert a HARDI data set into a multi-shell or DSI data set. Theoretically, a HARDI data set does not have the radial information that can differentiate slow diffusion from fast diffusion due to its uniform diffusion sensitization strength. Thus, some information loss is unavoidable in this process and the conversion from HARDI to multi-shell or DSI data set cannot fully recover this missing information.

Due to this missing information, we cannot remove the false fibers caused by partial volume of free water in the converted HARDI data. This fundamentally limits what types of analysis can be applied to data already collected using a single-shell scheme. We should point out, however, that although the diffusion metrics cannot be directly compared due to different *b*-values and diffusion times, we have shown a strong correlation between the converted and original HARDI data. Thus, it is still feasible to aggregate data sets using a multi-site analysis approach, in which the acquisition difference can be modeled as a site difference (e.g., a categorical variable indicating scanner environment in a regression analysis). For analysis concerning mostly fiber orientation (e.g., graph theoretical analysis), the scheme conversion can be used since the fiber orientations are largely identical. Lastly, the convertibility itself may shade a light on the redundancy issue of diffusion acquisition and help develop an optimal sampling strategy in the near future.

In conclusion, we show that schemes with multiple *b*-values, such as multi-shell acquisition and DSI, can be converted to their corresponding single-shell HARDI images with high fidelity. The diffusion signals, anisotropy values, and diffusivity measurements derived from converted HARDI strongly predict those of the original HARDI. The global network measures show less than 2% difference, whereas local nodal measures show 4~7% difference. The converted HARDI data achieves an angular resolution comparable to the original HARDI data. These results give a solid support for connectomic studies to continue using multi-shell or DSI acquisitions over the more popular DTI and HARDI approaches and highlights a utility for comparing these data sets to previously collected single-shell experiments.

## Author contributions

FY conducted the analysis and wrote the paper. TV acquired image data and wrote the paper.

### Conflict of interest statement

The authors declare that the research was conducted in the absence of any commercial or financial relationships that could be construed as a potential conflict of interest.

## References

[B1] Abhinav K. Yeh F. C. Pathak S. Suski V. Lacomis D. Friedlander R. M.. (2014). Advanced diffusion MRI fiber tracking in neurosurgical and neurodegenerative disorders and neuroanatomical studies: a review. Biochim. Biophys. Acta 1842, 2286–2297. 10.1016/j.bbadis.2014.08.002 25127851

[B2] Bammer R. Markl M. Barnett A. Acar B. Alley M. T. Pelc N. J.. (2003). Analysis and generalized correction of the effect of spatial gradient field distortions in diffusion-weighted imaging. Magn. Reson. Med. 50, 560–569. 10.1002/mrm.10545 12939764

[B3] Basser P. J. Mattiello J. LeBihan D. (1994). Estimation of the effective self-diffusion tensor from the NMR spin echo. J. Magn. Reson. B 103, 247–254. 10.1006/jmrb.1994.1037 8019776

[B4] Basser P. J. Pierpaoli C. (1996). Microstructural and physiological features of tissues elucidated by quantitative-diffusion-tensor MRI. J. Magn. Reson. B 111, 209–219. 10.1006/jmrb.1996.0086 8661285

[B5] Behrens T. E. Woolrich M. W. Jenkinson M. Johansen-Berg H. Nunes R. G. Clare S.. (2003). Characterization and propagation of uncertainty in diffusion-weighted MR imaging. Magn. Reson. Med. 50, 1077–1088. 10.1002/mrm.10609 14587019

[B6] Behrens T. E. Berg H. J. Jbabdi S. Rushworth M. F. Woolrich M. W. (2007). Probabilistic diffusion tractography with multiple fibre orientations: what can we gain? Neuroimage 34, 144–155. 10.1016/j.neuroimage.2006.09.018 17070705 PMC7116582

[B7] Bullmore E. Sporns O. (2009). Complex brain networks: graph theoretical analysis of structural and functional systems. Nat. Rev. Neurosci. 10, 186–198. 10.1038/nrn2575 19190637

[B8] Callaghan V. P. T. (1991). Principles of Nuclear Magnetic Resonance Microscopy. Oxford: Oxford Science Publications; Clarendon Press.

[B9] Daducci A. Canales-Rodríguez E. J. Descoteaux M. Garyfallidis E. Gur Y. Lin Y. C.. (2014). Quantitative comparison of reconstruction methods for intra-voxel fiber recovery from diffusion MRI. IEEE Trans. Med. Imaging 33, 384–399. 10.1109/TMI.2013.2285500 24132007

[B10] Dell'Acqua F. Scifo P. Rizzo G. Catani M. Simmons A. Scotti G.. (2009). A modified damped Richardson-Lucy algorithm to reduce isotropic background effects in spherical deconvolution. Neuroimage 49, 1446–1458. 10.1016/j.neuroimage.2009.09.033 19781650

[B11] Fan Q. Witzel T. Nummenmaa A. Van Dijk K. R. Van Horn J. D. Drews M. K.. (2016). MGH-USC Human Connectome Project datasets with ultra-high b-value diffusion MRI. Neuroimage 124, 1108–1114. 10.1016/j.neuroimage.2015.08.075 26364861 PMC4651764

[B12] Hui E. S. Cheung M. M. Chan K. C. Wu E. X. (2010). B-value dependence of DTI quantitation and sensitivity in detecting neural tissue changes. Neuroimage 49, 2366–2374. 10.1016/j.neuroimage.2009.10.022 19837181

[B13] Jovicich J. Czanner S. Greve D. Haley E. van der Kouwe A. Gollub R.. (2006). Reliability in multi-site structural MRI studies: effects of gradient non-linearity correction on phantom and human data. Neuroimage 30, 436–443. 10.1016/j.neuroimage.2005.09.046 16300968

[B14] Kuo L. W. Chen J. H. Wedeen V. J. Tseng W. Y. (2008). Optimization of diffusion spectrum imaging and q-ball imaging on clinical MRI system. Neuroimage 41, 7–18. 10.1016/j.neuroimage.2008.02.016 18387822

[B15] Le Bihan D. Johansen-Berg H. (2012). Diffusion MRI at 25: exploring brain tissue structure and function. Neuroimage 61, 324–341. 10.1016/j.neuroimage.2011.11.006 22120012 PMC3683822

[B16] McNab J. A. Edlow B. L. Witzel T. Huang S. Y. Bhat H. Heberlein K.. (2013). The Human Connectome Project and beyond: initial applications of 300 mT/m gradients. Neuroimage 80, 234–245. 10.1016/j.neuroimage.2013.05.074 23711537 PMC3812060

[B17] Papinutto N. D. Maule F. Jovicich J. (2013). Reproducibility and biases in high field brain diffusion MRI: an evaluation of acquisition and analysis variables. Magn. Reson. Imaging 31, 827–839. 10.1016/j.mri.2013.03.004 23623031

[B18] Pullens P. Roebroeck A. Goebel R. (2010). Ground truth hardware phantoms for validation of diffusion-weighted MRI applications. J. Magn. Reson. Imaging 32, 482–488. 10.1002/jmri.22243 20677281

[B19] Sotiropoulos S. N. Jbabdi S. Xu J. Andersson J. L. Moeller S. Auerbach E. J.. (2013). Advances in diffusion MRI acquisition and processing in the Human Connectome Project. Neuroimage 80, 125–143. 10.1016/j.neuroimage.2013.05.057 23702418 PMC3720790

[B20] Tournier J. D. Calamante F. Connelly A. (2007). Robust determination of the fibre orientation distribution in diffusion MRI: non-negativity constrained super-resolved spherical deconvolution. Neuroimage 35, 1459–1472. 10.1016/j.neuroimage.2007.02.016 17379540

[B21] Tournier J. D. Mori S. Leemans A. (2011). Diffusion tensor imaging and beyond. Magn. Reson. Med. 65, 1532–1556. 10.1002/mrm.22924 21469191 PMC3366862

[B22] Tournier J. D. Yeh C. H. Calamante F. Cho K. H. Connelly A. Lin C. P. (2008). Resolving crossing fibres using constrained spherical deconvolution: validation using diffusion-weighted imaging phantom data. Neuroimage 42, 617–625. 10.1016/j.neuroimage.2008.05.002 18583153

[B23] Tuch D. S. (2004). Q-ball imaging. Magn. Reson. Med. 52, 1358–1372. 10.1002/mrm.20279 15562495

[B24] Tuch D. S. Reese T. G. Wiegell M. R. Makris N. Belliveau J. W. Wedeen V. J. (2002). High angular resolution diffusion imaging reveals intravoxel white matter fiber heterogeneity. Magn. Reson. Med. 48, 577–582. 10.1002/mrm.10268 12353272

[B25] Tzourio-Mazoyer N. Landeau B. Papathanassiou D. Crivello F. Etard O. Delcroix N.. (2002). Automated anatomical labeling of activations in SPM using a macroscopic anatomical parcellation of the MNI MRI single-subject brain. Neuroimage 15, 273–289. 10.1006/nimg.2001.0978 11771995

[B26] Wedeen V. J. Hagmann P. Tseng W. Y. Reese T. G. Weisskoff R. M. (2005). Mapping complex tissue architecture with diffusion spectrum magnetic resonance imaging. Magn. Reson. Med. 54, 1377–1386. 10.1002/mrm.20642 16247738

[B27] Wedeen V. J. Rosene D. L. Wang R. Dai G. Mortazavi F. Hagmann P.. (2012). The geometric structure of the brain fiber pathways. Science 335, 1628–1634. 10.1126/science.1215280 22461612 PMC3773464

[B28] Yeh F. C. Tseng W. Y. (2013). Sparse solution of fiber orientation distribution function by diffusion decomposition. PLoS ONE 8:e75747. 10.1371/journal.pone.0075747 24146772 PMC3795723

[B29] Yeh F. C. Verstynen T. D. Wang Y. Fernández-Miranda J. C. Tseng W. Y. (2013). Deterministic diffusion fiber tracking improved by quantitative anisotropy. PLoS ONE 8:e80713. 10.1371/journal.pone.0080713 24348913 PMC3858183

[B30] Yeh F. C. Wedeen V. J. Tseng W. Y. (2010). Generalized q-sampling imaging. IEEE Trans. Med. Imaging 29, 1626–1635. 10.1109/TMI.2010.2045126 20304721

[B31] Yeh F. C. Wedeen V. J. Tseng W. Y. (2011). Estimation of fiber orientation and spin density distribution by diffusion deconvolution. Neuroimage 55, 1054–1062. 10.1016/j.neuroimage.2010.11.087 21232611

